# Robust phase determination in complex solid solutions using diffuse multiple scattering

**DOI:** 10.1107/S1600576723004120

**Published:** 2023-06-12

**Authors:** A. G. A. Nisbet, M. G. Cain, T. Hase, P. Finkel

**Affiliations:** a Diamond Light Source, Harwell Science & Innovation Campus, Harwell OX11 0DE, United Kingdom; b Electrosciences Ltd, Farnham, Surrey GU9 9QT, United Kingdom; c University of Warwick, Coventry CV4 7AL, United Kingdom; d US Naval Research Laboratory, Washington, District of Columbia 20375, USA; HPSTAR and Harbin Institute of Technology, People’s Republic of China

**Keywords:** morphotropic, piezoelectric, diffuse multiple scattering, phase determination, complex solid solutions, fingerprinting, Kossel lines

## Abstract

A novel methodology is presented for identifying and distinguishing between structural phases in multi-phasic systems, such as piezoelectric materials like PMN–PT [Pb(Mg_1/3_Nb_2/3_)O_3_–PbTiO_3_], PIN–PMN–PT [Pb(In_1/2_Nb_1/2_)O_3_–Pb(Mg_1/3_Nb_2/3_)O_3_–PbTiO_3_] and PZT [Pb(Zr,Ti)O_3_], using diffuse multiple scattering and Kossel line diffraction techniques. The method exploits the splitting of triple line intersections from special coplanar reflections combined with logical constraints to generate a splitting fingerprint for robust crystallographic phase determination and discrimination.

## Abstract 2

A new fingerprinting methodology is presented for phase discrimination in complex solid solutions near the morphotropic phase boundary.

## 1. Introduction

Piezoelectric materials, such as PMN–PT [Pb(Mg_1/3_Nb_2/3_)O_3_–PbTiO_3_], PIN–PMN–PT [Pb(In_1/2_Nb_1/2_)O_3_–Pb(Mg_1/3_Nb_2/3_)O_3_–PbTiO_3_] and PZT [Pb(Zr,Ti)O_3_], exhibit the giant piezoelectric effect at compositions near the morphotropic phase boundary (Park & Shrout, 1997 ▸; McLaughlin *et al.*, 2004 ▸). This is a region on the compositional phase diagram where there is a near degeneracy of crystallographic phases, resulting in the coexistence of multiple phases and domains. This can be problematic for conventional high-resolution X-ray scattering techniques because the movement required to measure multiple reflections essential for structural determination results in averaging over multiple domains. Here, we present a novel methodology for distinguishing between phases and robustly determining their lattice parameters using diffuse multiple scattering (DMS). Nisbet *et al* (2015 ▸) present an explanation of DMS with an emphasis on non-coplanar triple intersections; however, the methodology presented here relies on the splitting of coplanar triple intersections and is applicable to other *K*-line techniques. PIN–PMN–PT under applied mechanical stress and electrical polling has been used as a demonstration because the crystal exhibits two phases at low stress, which transition to a single phase at high stress (Patterson *et al.*, 2020 ▸; Finkel *et al.*, 2022 ▸).

**Figure 1:**
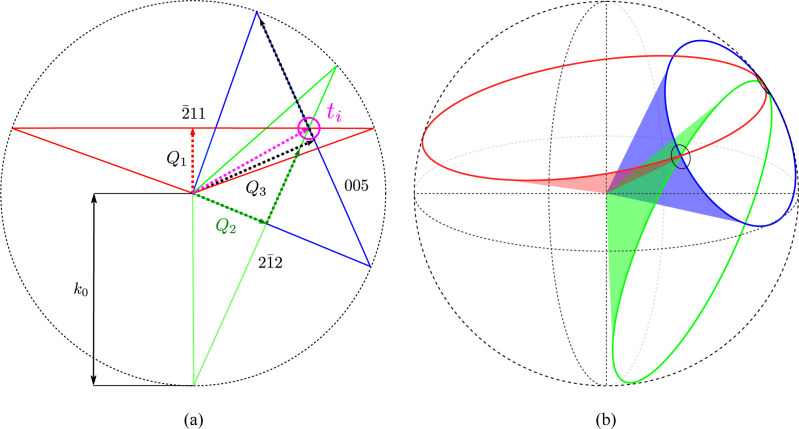
(*a*) Three coplanar reflections for a cubic crystal: 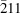 , 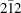 and 005, coloured red, green, and blue, respectively. The **Q** vectors are represented by dashed arrows, and their lengths are equal to *d* ^−1^/2, or simply *d* ^−1^ because the denominator cancels out. The triple intersection is highlighted by a small magenta circle (*t*_i_). The radius of the large circle is equal to *k*_0_, which is equal to λ^−1^. The red, green and blue solid lines show the cone projections along **Q**_1_ × **Q**_*i*_. (*b*) A three-dimensional representation of (*a*) to show that the intersection occurs out of the plane common to *Q*_1_, *Q*_2_ and *Q*_3_.

In DMS and *K*-line diffraction, a divergent source of X-rays results in constructively interfering X-rays emerging as cones, producing elliptical lines as they are projected onto the detector plane. Triple intersections can arise at specific energies for a given set of non-coplanar reflections (Lonsdale, 1947 ▸). This also provides a powerful technique for crystallographic phase determination, which is the subject of an earlier paper (Nisbet *et al.*, 2021 ▸). The current article focuses on triple intersections from coplanar reflections, which Lonsdale referred to as ‘geometrically inevitable’ (Lonsdale, 1947 ▸). This is presented diagrammatically in Fig. 1 ▸ for the 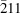 , 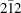 and 005 coplanar reflections.

The formal condition for a coplanar triple intersection is given by equation (1) (Harris, 1975 ▸): 

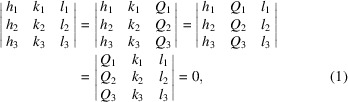

where *Q*_*i*_ in this case is 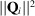 . There is a subset of coplanar triple intersections that occur when the first reflection is perpendicular to the second reflection and the tertiary reflection is the sum of the first two. While this is not general, it gives a useful rule of thumb for the condition above.

These intersections are not unique to specific energies and can be selected for their sensitivity to specific symmetry changes in the crystal. For example, a triple intersection produced by the 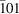 , 101 and 002 reflections would be sensitive to a change from cubic to tetragonal symmetry because a change in the *a* or *c* directions would result in one of the determinants in equation (1) being non-zero. A change in *b*, however, would not result in splitting, because all of the determinants would remain zero. On the other hand, a triple intersection produced by the 002, 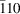 and 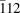 reflections is insensitive to changes in *a*, *b* or *c* but is sensitive to rhombohedral or monoclinic distortions. In the case of a rhombohedral distortion, a triple intersection will occur for these reflections. However, if the lines are indexed as 002, 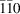 and 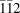 , for example, the intersection will be split.

The observation of splitting at carefully selected triple intersections means a splitting fingerprint can be constructed, and then a relatively simple procedure can be followed to identify the crystallographic phases present and even distinguish between multiple phases. This is particularly useful in complex solid solutions where the crystallographic differences are subtle.

## 2. Experimental

**Figure 2:**
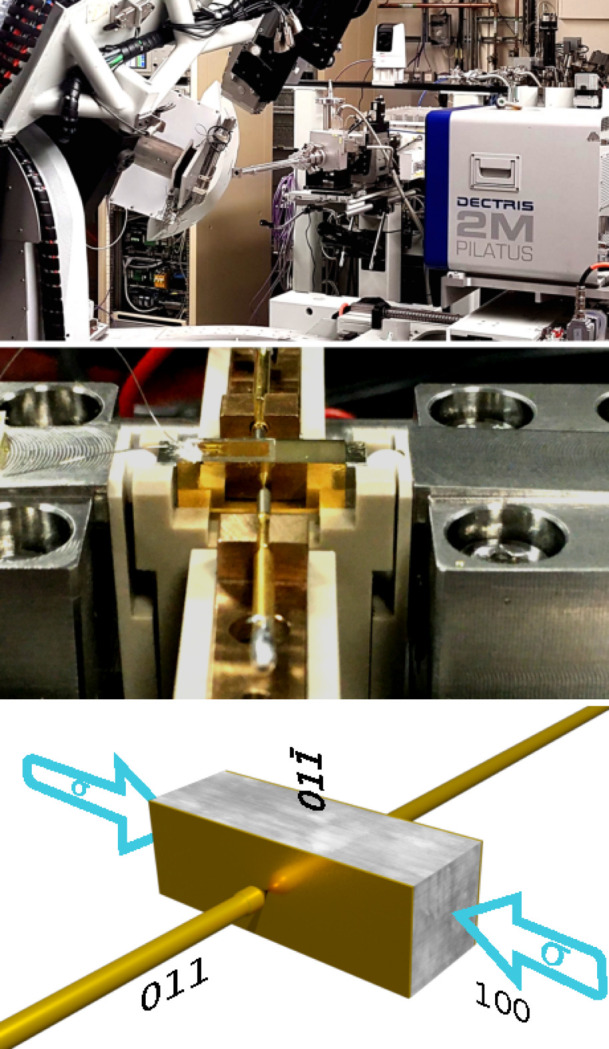
The experimental setup on the I16 beamline at Diamond Light Source. The detector is positioned perpendicular to the incident beam and parallel to the incident polarization (top). The Model ES1500 multifunctional stress–strain system with high-voltage terminals is also shown (bottom).

**Figure 3:**
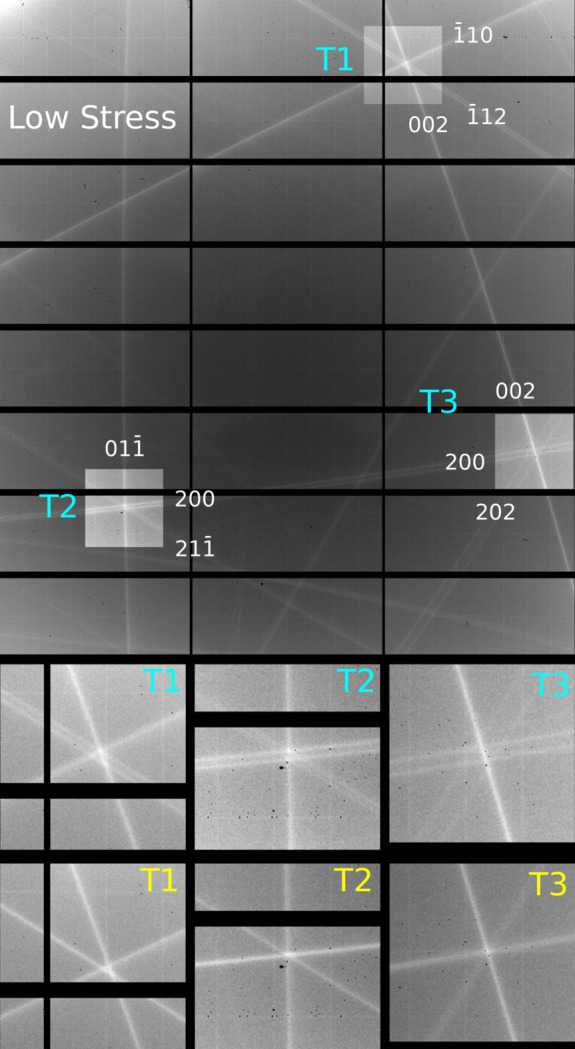
The large central image is a single exposure taken using a Pilatus 2M detector at the (1.568 1.214 3.976) non-integer reflection at 9.6325 keV at low mechanical stress (−6.8 MPa). Three regions are highlighted and enlarged for clarity. T1, T2 and T3 (cyan) represent the line intersections of coplanar reflections. T1, T2 and T3 (yellow) represent the same line intersections at high stress (−24 MPa).

The [011] poled PIN–PMN–PT, 12 × 4 × 4 mm, sample was placed in a multifunctional stress–strain system (Model ES1500 by Electrosciences Ltd) and mounted on a Newport kappa-geometry diffractometer on the I16 beamline (Collins *et al.*, 2010 ▸) at Diamond Light Source (Fig. 2 ▸). The sample was prepared with gold electrodes and connected to a Trek high-voltage power supply. The sample was orientated using two Bragg reflections to build a **UB** orientation matrix (Busing & Levy, 1967 ▸), where the **B** matrix transforms a given *hkl* to an orthonormal coordinate system fixed in the crystal and the **U** matrix is a rotation matrix that rotates the crystal’s reference frame to that of the diffractometer. This simplifies the initial indexing of the lines. The sample was orientated to a non-integer *hkl* (1.5697, 1.2153, 3.9792) at a ψ value (azimuthal angle) of 16° with an azimuthal reference of 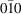 in the pseudo-cubic setting to scatter in the plane parallel to the incident polarization vector. A non-integer reflection was used to minimize the signal from direct Bragg reflections. A 2M Pilatus detector was positioned at a distance of 1071 mm from the sample and perpendicular to the incident beam to minimize background scatter. The beam size was 180 µm in the horizontal direction and 20 µm in the vertical direction, and the image acquisition time was 200 s. The sample was stressed along the [100] direction from −6.8 to −24 MPa and back again in 1.323 MPa steps, with a cycled electric field of 0 to 250 kV mm^−1^ and back to 0 kV mm^−1^ in steps of 50 kV mm^−1^ applied along the [011] direction. Initially, two phases were present under low stress, indicated by the split lines in Fig. 3 ▸ (top panel), evolving to a single phase at high stress. The system reverts back to two phases at low stress.

## 3. Analysis

**Figure 4:**
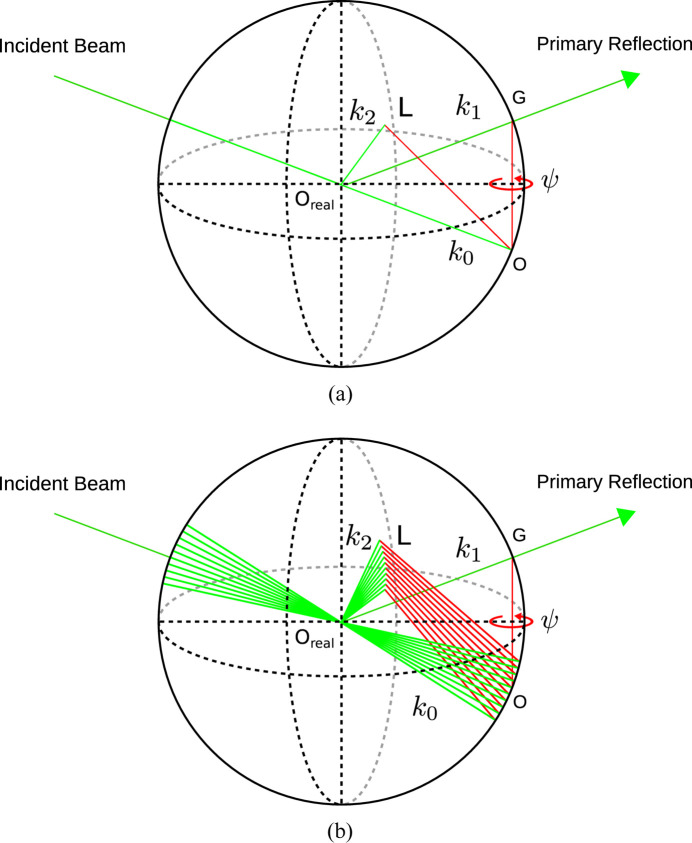
Ewald sphere construction of (*a*) MS and (*b*) DMS.

DMS can be described by an extension of the multiple scattering (MS) geometry. A geometrical representation of the MS condition is conveniently described using Ewald construction [Fig. 4 ▸(*a*)].

The radius of the sphere is given by the reciprocal of the wavelength, **G** is the primary scattering vector, **L** is a secondary scattering vector, **k**_0_ is the incident beam vector, **k**_1_ is the primary reflected beam vector, and **k**_2_ and **k**_*n*_ are the secondary beam vectors. O_real_ is the real-space origin, O is the reciprocal-space origin and ψ is the azimuthal angle defined with respect to an azimuthal reference vector, which will lie in the plane defined by **k**_0_ and **k**_1_ and away from the incident beam when ψ is equal to zero. When **L** is rotated around **G** and intercepts the sphere, MS occurs. The geometry can easily be extended for DMS by replacing the primary scattering vector with a continuum of co-aligned vectors. This is equivalent to allowing the primary scattering vector to be a diffuse source, which in this case arises from defects in the sample (Nisbet *et al.*, 2015 ▸). Fig. 4 ▸(*b*) shows how scaling **G** changes the reciprocal-space origin and thus the azimuthal interception angle of **L**. Converting to spherical coordinates, using the non-integer Bragg angle as the polar angle, ψ as the azimuthal angle and *k*_0_ as the vector lengths, is equivalent to calculating diffraction conics.

A benefit of calculating DMS this way is that the θ range can be limited to the detector acceptance angle. The scalar equation for a plane can be used to define the detector plane and determine the coordinates at which the DMS vectors intercept the detector. These can then be binned appropriately to generate an image of the DMS lines or create regions of interest for fitting algorithms (Nisbet *et al.*, 2021 ▸).

### 3.1. Indexing

Using two non-parallel reflections to build a **UB** matrix is routine on diffraction beamlines. This allows samples to be conveniently orientated using reciprocal-space vectors. Typically, for a DMS measurement, a non-integer reflection will be selected. Ideally, this will have a 2θ angle of around 90° with the scattering plane parallel to the incident beam polarization, which significantly reduces the scattering background. A simulated DMS pattern can be calculated using the experimental geometry and directly overlaid on the detector image. Setting a relatively high structure-factor threshold (95th percentile) for a low-level cut-off eases the comparison between simulated and experimental DMS lines, allowing the lines to be indexed straightforwardly via manual selection.

### 3.2. Constructing a truth table

The T3 triple intersection is composed of the 002, 200 and 202 reflections. The 002 and 200 reflections are perpendicular to one another. This means that there are 12 possible sets of equivalent indices, which reduces for lower-symmetry crystals. This re-indexing can be done by applying each of the 12 transformation matrices in Table 1 ▸.

**Figure 5:**
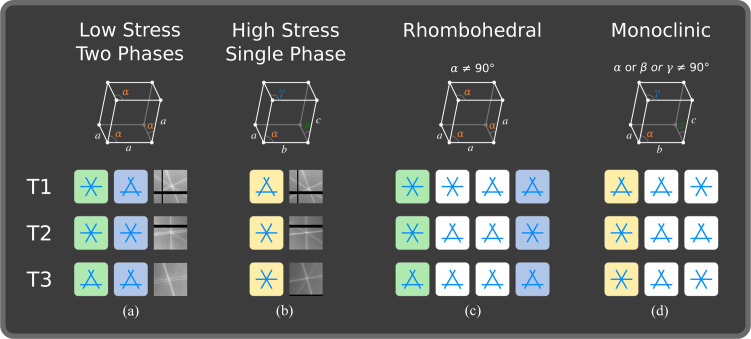
(*a*) Two splitting fingerprints measured at low stress. (*b*) The observed fingerprint measured at high stress. (*c*) Calculated fingerprints for the four inequivalent settings of the rhombohedral phase. (*d*) Calculated fingerprints for the three inequivalent settings of the monoclinic phase.

The reflections in T1, T2 and T3 intersections are multiplied by each transformation matrix and tested against equation (1) for a rhombohedral distortion to determine if the intersections are split. For example, as mentioned above, the 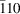 , 002 and 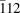 reflections without transformation satisfy equation (1) when a rhombohedral distortion is applied. However, by changing the indexing according to the first matrix in row 4 of Table 1 ▸, the reflections become 110, 002 and 112, and equation (1) is no longer satisfied. The same logic was applied to cubic, tetragonal and orthorhombic crystals, which all give a single truth table of [1,1,1]; while triclinic, assuming at least two angles deviate from 90° and are not equal, gives a single truth table of [0,0,0]. The truth tables for rhombohedral and monoclinic crystals are presented pictorially in Fig. 5 ▸.

### 3.3. Phase identification

Five lines can be seen at T3 in Fig. 3 ▸. These are produced by two phases and can be indexed as the 002, 200 and 202 reflections. The absence of closed triple intersections immediately eliminates the possibility of cubic, tetragonal and orthorhombic phases being present. This is because changes in *a*, *b* or *c*, or any combination thereof, cannot split the intersections produced by this combination of coplanar reflections. T1, comprising the 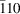 , 002 and 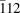 reflections, shows a split intersection and a closed intersection. Of course, a different assignment of the lines could mean that there were two split intersections. While this is an unlikely occurrence, it is easily checked with DMS by changing the incident wavelength, which can split the intersections formed by lines from neighbouring phases. T2 shows two closed intersections composed of the 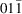 , 200 and 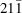 reflections. Now we can construct the table for the low-stress phase shown in Fig. 5 ▸(*a*). The two sets of lines merge at high stress. T1 is split, while T2 and T3 are closed. As before, the splitting of just one of the intersections eliminates the possibility of cubic, tetragonal and ortho­rhombic phases. Identifying these phases is a simple process of comparing the table constructed directly from the data with the tables presented in Figs. 5 ▸(*c*) and 5 ▸(*d*). This can be done independently of the experimental geometry.

Fig. 5 ▸ shows the splitting for the four unique settings for the rhombohedral crystal system [Fig. 5 ▸(*c*)] and the three unique settings for the monoclinic system [Fig. 5 ▸(*d*)]. The low-stress phases can be explained by the green and blue columns (columns 1 and 4), indicating that both phases are rhombohedral, or two domains of the same phase. Following the same logic, the splitting pattern for the high-stress phase matches the yellow column (column 1), indicating that it is monoclinic. Once the phases have been identified, the lines can be assigned to their respective phases and used to refine the lattice parameters to a precision of 5 × 10^−5^Å (Lonsdale, 1947 ▸; Nisbet *et al.*, 2021 ▸).

## 4. Conclusions

We have demonstrated a new methodology for discriminating between phases in complex solid solutions. A splitting fingerprint over multiple triplets can be generated. This reduces the phase identification to the simple task of using a lookup table. The technique has been developed for DMS but is applicable to Kossel lines (Kossel, 1935 ▸) and pseudo-Kossel lines (Imura, 1954 ▸).

## Figures and Tables

**Table 1:** Truth table for a rhombohedral crystal based on T1, T2 and T3 calculated from equation (1) A value of 1 means there is a triple intersection. A value of 0 means there is a split intersection. The three transformation matrices in each row are equivalent and give the same vector representation in the truth table.

Transformation matrices	T1 T2 T3 (rhombohedral)
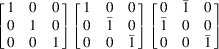	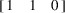
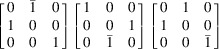	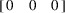
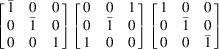	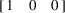
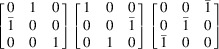	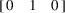
